# Transcriptome Profiling Reveals Candidate Genes Related to Stipe Gradient Elongation of *Flammulina filiformis*

**DOI:** 10.3390/jof9010064

**Published:** 2022-12-31

**Authors:** Junjie Yan, Zongjun Tong, Xing Han, Ying Gan, Yuanyuan Liu, Jie Chen, Xinlian Duan, Junbin Lin, Bingcheng Gan, Baogui Xie

**Affiliations:** 1 Institute of Urban Agriculture, Chinese Academy of Agricultural Sciences, Chengdu 610000, China; 2 Mycological Research Center, College of Life Sciences, Fujian Agriculture and Forestry University, Fuzhou 350002, China; 3 Chengdu Agricultural Science and Technology Center, Chengdu 610000, China

**Keywords:** needle mushroom, stipe elongation, RNA-seq, ribosome, long-chain fatty acid

## Abstract

Stipe gradient elongation is an important and remarkable feature in the development of most mushroom fruiting bodies. However, its molecular mechanism has rarely been described. Here, the decreasing trend of stipe elongation and increasing trend of cell length in a gradient from the top to the base of the stipe were determined in a model basidiomycete mushroom: *Flammulina filiformis*. According to RNA-seq results, 1409 differentially expressed genes (DEGs) were identified among elongation region (ER), transition region (TR), and stable region (SR) samples, including 26 transcription factors (TFs). Based on Short Time-series Expression Miner (STEM) clustering of DEGs, clusters 1 and 3, with obvious expression trends that were consistent with or in contrast to the elongation rate, were screened. The cluster 1 DEGs were mainly involved in the GO cellular component category and KEGG genetic information processing class; however, the cluster 3 DEGs were mainly involved in metabolic processes. Furthermore, qRT-PCR confirmed that key genes of the long-chain fatty acid synthesis pathway were involved in stipe gradient elongation and regulated by NADPH oxidase-derived ROS signaling molecules. These findings provide an essential basis for understanding the molecular mechanism of stipe gradient elongation.

## 1. Introduction

The shaping of fruiting body in mushroom represents the most complex developmental process known in the fungal kingdom, and stipe elongation is one of the key features of fruiting body development [1,2]. It was demonstrated that stipe elongation is non-uniform in many mushrooms, such as *Agaricus bisporus*, *Flammulina filiformis*, and *Coprinopsis cinerea* [3,4,5]. The elongation rate decreases in a gradient from the apex to the base of the stipe in most basidiomycete mushrooms [2,6]. Several studies have suggested that the main driving force of stipe elongation is manifold cell elongation [7].

Two long stipe mushroom species, *C. cinerea* and *F. filiformis*, have been used as model systems to study the stipe elongation mechanism. In *C. cinerea,* the stipe can be divided into three regions: the apical 9 mm region elongates the most, followed by the middle 9 mm region with considerable elongation, and the 9 mm basal region no longer elongates during the development of the 27 mm long young fruiting body [4]. Niu et al. showed that the cell wall architecture varies in different stipe regions [8]. Several studies suggested that glucanase, chitinase, and β-glucan-degrading enzymes associated with cell wall extension are involved in stipe elongation regulation [9,10,11,12]. In addition, the genes encoding septin protein Cc.Cdc3 and MAPK homologue CcSakA were also suggested to have important roles in controlling stipe elongation in *C. cinerea* [13,14]. In *F. filiformis*, the fruiting body at a length of 8.5 cm was defined as the elongation stage; in this stage, the apical 1.5 cm showed a fast elongation rate, the 1.5–3 cm section showed a slow elongate rate, and the region below 3 cm showed no further elongation [6]. Multiple genes involved in stipe elongation regulation have been annotated based on gene expression pattern analysis, such as the genes encoding expansin-like protein [15], S-adenosylmethionine-dependent methyltransferase [16], cytochrome c peroxidase [17], and chromatin modifier protein [18]. Our recent study also demonstrated that NADPH oxidase and Mn-SOD driving ROS signaling molecule distribution have key roles in stipe gradient elongation of *F. filiformis* [6].

Transcriptomics based on RNAseq, which have been used to study the molecular mechanism of fruiting body development for several years, provide powerful tools to understand the biology of mushrooms [19]. Based on gene expression profiling, Tao et al. found that the stipe development of *Volvariella volvacea* is a fairly complex process, mainly by changes in expression levels [20]. Liu et al. identified conserved stipe-specific expression patterns during sexual development in *F. filiformis* by transcriptome data analysis [21]. Yan et al., based on comparative transcriptomics, suggested that a high CO_2_ concentration inhibits early pileus expansion of *F. filiformis* by decreasing cell division control pathways [22]. The transcriptomics sequencing method has also been extensively used to reveal the molecular mechanism of fruiting body development in multiple mushroom species [23,24,25,26]. However, most omics studies treated the stipe as an integral part. The gene expression patterns of different stipe regions at the genome level have not been studied, and the molecular mechanism of stipe gradient elongation has yet to be elucidated.

As a commercial and factory cultivation mushroom, *F. filiformis* is widely cultivated and consumed in Asian countries due to its high nutritional value and desirable taste [27]. The long stipe and small pileus represent the typical fruiting body morphology of cultivated *F. filiformis*, making it an ideal model for investigating the stipe elongation mechanism [6]. To better understand the molecular mechanism of stipe elongation, the transcriptomes of three elongated stipe regions were analyzed and the genes correlated with the stipe elongation rate were examined. This transcriptomic information could facilitate our understanding of the genetic and molecular mechanisms of stipe gradient elongation in *F. filiformis.* Furthermore, the results are also a prerequisite for the breeding of varieties and achieving the precise control of stipe morphology.

## 2. Materials and Methods

### 2.1. Microorganism and Cultivation Conditions

*Flammulina filiformis* Fv01 (commercial white dikaryotic strain mated by 2 monokaryotic strains, Fv01-N and Fv01–10) was obtained from the Fujian Edible Fungi Germplasm Resource Collection Center of China. The fruiting bodies were cultivated with substrate medium at 10 °C, according to our previous method [6].

### 2.2. Stipe Elongation Rate Measurement and Cell Length Detection

Based on Yan et al.’s (2022) results, fruiting bodies in the elongation stage with 8.5 cm stipe length were selected based a previous study [6]. The elongation rate of different regions was measured within 12 h growth time at 10 °C. Then, 3 mm long fresh segments around the 1, 2, 4, and 6 cm stipe regions were collected and dissected into small pieces according to Shioya et al.’s (2013) methods [13], the images of stipe cells were taken under a Leica DM4B microscope (Leica Corporation, Wetzlar, Germany), and cell length between two adjacent septums were examined.

### 2.3. Sample Collection, RNA Extraction, Library Construction, and Sequencing

Different stipe regions were collected by using the paired sampling method. RNA extraction and library construction were performed according to previous methods [22]. In detail, total RNA was isolated from frozen samples using an E.Z.N.A.™ Plant RNA Kit (Omega, Stamford, CT, USA) according to the manufacturer’s protocol. Then, RNA was quantified using a NanoND-1000 spectrophotometer (NanoDrop Technologies, Wilmington, DE, USA). The RNA libraries were prepared using an NEBNext Ultra RNA Library Prep Kit for Illumina (NEB, Ipswich, MA, USA) following the manufacturer’s recommendations.

The complementary DNA (cDNA) libraries were sequenced on an Illumina HiSeq X platform (Illumina Inc., San Diego, CA, USA) at Novogene Co., Ltd. (Tianjin, China), and 150 bp paired-end reads were generated.

### 2.4. RNA-Sequencing Data Analysis

Clean reads were obtained after processing to remove adapters, and paired reads were removed if one of them contained more than 10% poly-Ns or more than 50% low-quality base sequences. Then, the clean reads were aligned to the *F. filiformis* strain Fv01–10 genome (accession: PRJNA769814) [6] using HISAT2 with default parameters [28]. Differential expression analysis was performed using StringTie v.1.3.3 with default parameters [29]. The gene expression levels were expressed as fragments per kilobase per million reads (FPKM). Genes with 0 FPKM in all samples were excluded from the study, and differentially expressed genes (DEGs) were identified using paired *t*-tests with a *p*-value cutoff of 0.05 and a fold-change (FC) threshold of 1.2. Gene Ontology (GO; http://www.geneontology.org, accessed on 10 August 2022) and Kyoto Encyclopedia of Genes and Genomes (KEGG; https://www.genome.jp/kegg/, accessed on 2 October 2022) were used for gene function annotation. The iTAK program (http://itak.feilab.net/cgi-bin/itak/online_itak.cgi, accessed on 2 October 2022) was used for transcription factor annotation (e-value was set as 1 × 10^−5^) [30]. The bioinformatics analysis, including volcano plot, Venn diagram, cluster analysis, and heat map, was performed on BMKCloud nets (http://www.biocloud.net/). The gene expression pattern analysis was performed by Short Time-series Expression Miner (STEM) software on the OmicShare tools platform (www.omicshare.com/tools), with the threshold cluster number set to 8 and *p* < 0.05 used as the threshold for significance.

### 2.5. Gene Identification and Sequence Analysis

The gene sequences were obtained from the Fv01–10 genome and verified by Sanger sequencing; the PCR primers were designed using Primer Premier 6.0 (Table S1). Gene structure analysis was performed according to Yan et al.’s (2016) method by using strand-specific RNA-seq data of mixed samples of Fv01 mycelium and fruiting bodies [17,31]. The gene schematic diagram was drawn by the Gene Structure Display Server 2.0 (GSDS; http://gsds.cbi.pku.edu.cn/index.php, accessed on 18 August 2022) [32].

### 2.6. Quantitative Real-Time PCR (qRT-PCR)

The total RNA of samples was used for first-strand cDNA synthesis with TransScript One Step gDNA Removal and cDNA Synthesis SuperMix kits for qPCR (TransGen Biotech, Beijing, China). qRT-PCR was performed on a CFX96 multicolor real-time PCR detection system (Bio-Rad, Hercules, CA, USA) with TransStart Top Green qPCR SuperMix (TransGen Biotech, Beijing, China). Two internal control genes, glyceraldehyde-3-dehydrogenase (FfGPD) and Ras-related small GTPase (FfRAS), were used as reference genes [33]. The primers were designed using Primer Premier 6.0 (Table S2). Relative gene expression levels were calculated using 2^−∆∆Ct^ threshold cycle calculation [34].

### 2.7. Fatty Acid Profile Analysis

The dried samples were powdered to about 1 mm particle size for further analysis. Fatty acid isolation and derivatization were performed according to previous methods [35]. Fatty acid composition was detected by a 7890B-5977A gas chromatograph–mass spectrometer (GC/MS; Agilent Technologies, Santa Clara, CA, USA) with a DB-23 quartz capillary column (30 m × 320 um × 0.25 um; Agilent Technologies, Santa Clara, CA, USA), using helium as the carrier gas. The injector and detector temperatures were 250 and 230 °C, respectively. The oven temperature was set as follows: initiate at 50 °C and hold for 1 min, elevate to 175 °C at a rate of 25 °C/min, then to 230 °C at 4 °C/min. The injection volume was 1 μL, and duration of the analysis was 24.75 min. Detector voltage was set to 0.93 kV, and the EI ionization voltage of the metabolites was 70 eV. Mass spectra were recorded from 50 to 550 m/z.

Fatty acid identification was made by comparing the relative retention times of fatty acid methyl ester (FAME) peaks from samples with standards. The amount of FAME was quantitative by the external standard curve method, and the FAME values were converted into the respective fatty acid contents in μg per gram of DW.

### 2.8. Statistical Analysis

In this study, statistical analysis was performed by GraphPad Prism 6.0 (GraphPad Software, San Diego, CA, USA). Mean ± SEM was determined for each treatment group in the individual experiments, and significance tests were performed using Student’s *t*-tests or paired *t*-test. The Pearson correlation coefficient was analyzed by SPSS Statistics v20 software with a two-tailed test.

## 3. Results

### 3.1. Stipe Gradient Elongation Features of F. filiformis

**Figure 1:**
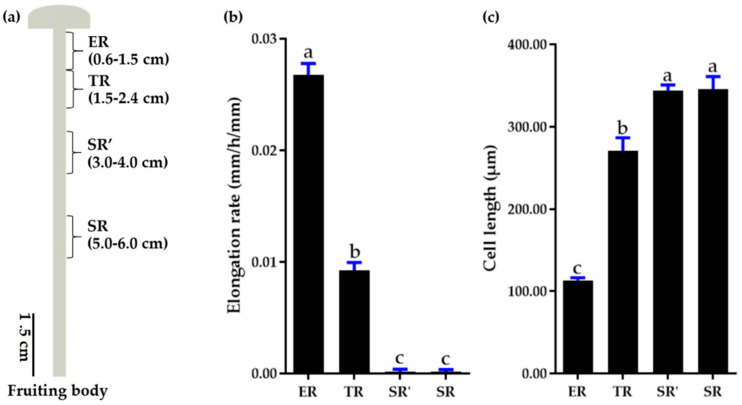
Stipe gradient elongation features of *F. filiformis*. (**a**) Stipe regions. ER, elongation region; TR, transition region; SR’ and SR, stable regions. (**b**) Elongation rate of different regions (*n* = 15). (**c**) Cell length of different regions (*n* = 4; value of each sample was average length of 50 cells). Different letters over columns denote significant differences (*p* < 0.05, paired *t* test).

In order to investigate the stipe gradient elongation features of *F. filiformis*, elongation stage fruiting bodies with a stipe approximately 8.5 cm in length were selected. An elongation region of 0.6–1.5 cm (ER), a transition region of 1.5–2.4 cm (TR), and stable regions of 3–4 cm (SR’) and 5–6 cm (SR) were marked according to our previous results (Figure 1a) [6]. As shown in Figure 1b, the ER region elongated rapidly, followed by TR with much slower elongation, and SR’ and SR regions did not elongate. In addition, the cells showed differences from elongation region to stable region. In detail, the cell length was the longest in SR’ and SR regions, followed by TR region, and the cells of ER region were significantly shorter than they were in the TR region (Figure 1c).

### 3.2. Transcriptional Changes in Different Stipe Regions of F. filiformis

To investigate the transcriptional change patterns during stipe elongation, three regions of ER, TR, and SR were collected for RNA-seq, applying three biological replicates for each region. As shown in Table S3, an average of 14,931,678 raw reads were generated per sample, yielding about 4.42 G clean bases on average for 9 samples (deposited in NCBI under BioprojectID PRJNA901539). For each of the 9 libraries, at least 94.03% of the clean reads had a quality score of Q30. The clean reads were compared with the genome sequence of *F. filiformis* Fv01–10 strain, and the mapping rate was higher than 95.5% for each sample.

**Figure 2:**
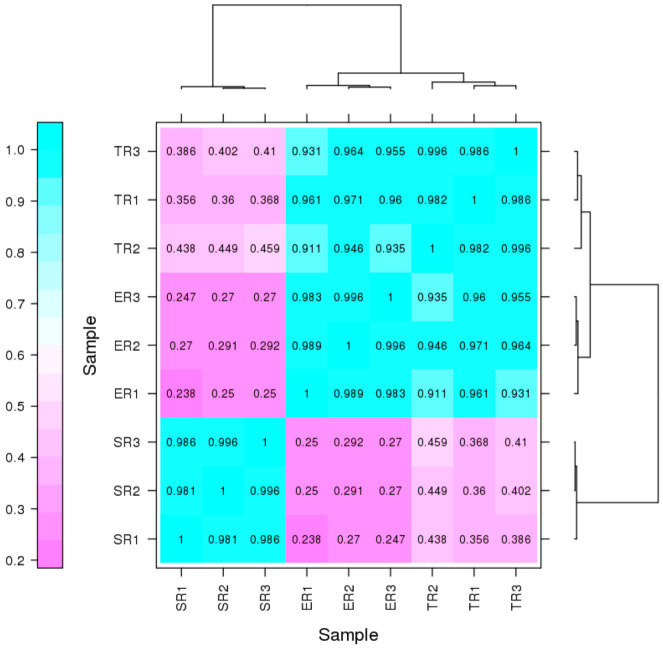
Pearson correlation coefficients for pairwise comparison of transcriptome data. ER, elongation region; TR, transition region; SR, stable region.

About 13,128 genes were found to be expressed in at least one sample after the mapping of clean reads against the *F. filiformis* genome (Table S4). Pearson’s correlation analysis between samples was performed (Figure 2), and the result shows r > 0.98 for replicate biological samples, indicating that the biological replicates were reliable. The SR group samples diverged from ER and TR groups, with Pearson’s correlation coefficient of about 0.250–0.292 for ER and 0.356–0.459 for TR, indicating that the biological process in the SR region may be very different from that in the other two regions. However, the samples showed little difference between ER and TR groups (Pearson correlation coefficient was about 0.911–0.971), suggest the similar function between these two regions.

### 3.3. Differentially Expressed Gene (DEG) Identification

**Figure 3:**
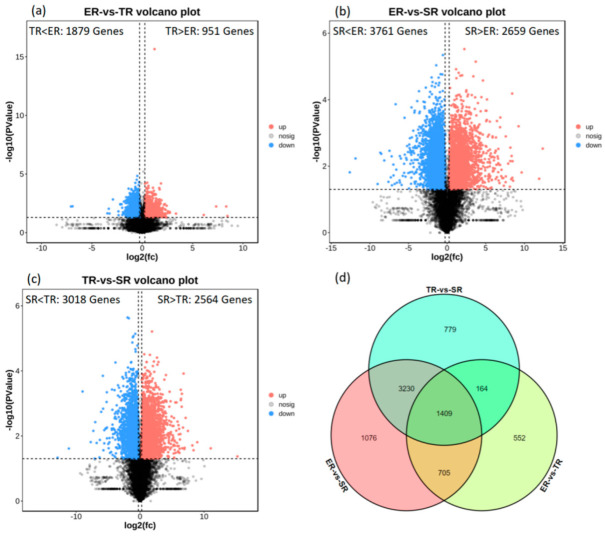
Analysis of DEGs in different samples. (**a**–**c**) Volcano plots illustrating DEGs between different stipe regions. (**d**) Venn diagram showing DEGs identified from comparison of ER, TR, and SR groups. ER, elongation region; TR, transition region; SR stable region.

To investigate the transcriptional divergence of the three stipe regions, DEGs were identified using the analytical thresholds of *p*-value < 0.05 and fold-change > 1.2. In ER vs. TR, about 951 upregulated and 1879 downregulated genes were detected (Figure 3a); in ER vs. SR, about 2659 upregulated and 3761 downregulated genes were detected (Figure 3b); and in TR vs. SR, about 2564 upregulated and 3018 downregulated genes were detected (Figure 3c). The results of comparative DEG analysis show that more genes were active in the high elongation rate region; however, more than 2500 upregulated genes in SR samples indicated that complex biological processes also occurred in the stable region. Venn diagram analysis showed that 1409 genes were expressed significantly differently among ER, TR, and SR groups (Figure 3d, Table S5).

**Figure 4:**
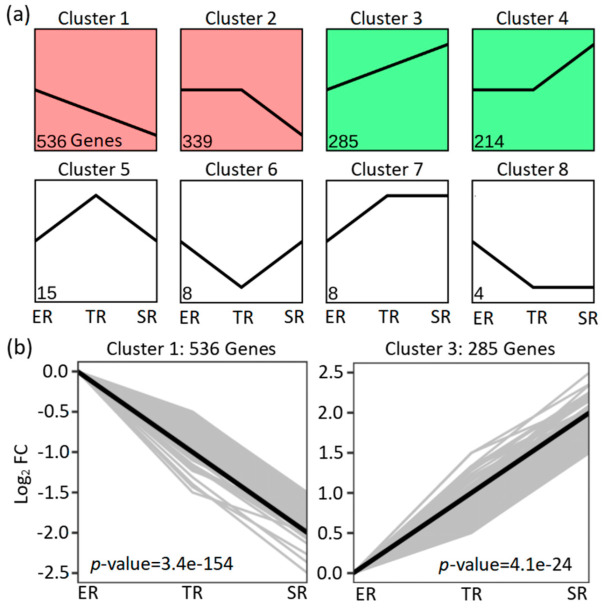
STEM analysis of 1409 DEGs based on significantly different genes in elongation region (ER), transition region (TR), and stable region (SR). (**a**) Eight expression clusters; (**b**) expression trends for genes in clusters 1 and 3.

In order to further analyze the patterns of gene expression across the three stipe regions, the expression profiles of 1409 significantly differently genes were obtained by using STEM software. Four significant (*p* < 0.05) gene expression clusters involving 1374 DEGs were screened (Figure 4a). In detail, cluster 1 (536 genes, *p*-value = 3.4 × 10^−154^) exhibited a clear gene expression trend of continuing to decrease from the apex to the base, consistent with the stipe elongation rate, while in cluster 3 (285 genes, *p*-value = 4.1 × 10^−24^) gene expression continued to increase from the apex to the base, in contrast to the elongation rate (Figure 4b, Table S6).

### 3.4. Transcription Factors (TFs) Annotation of DEGs

**Figure 5:**
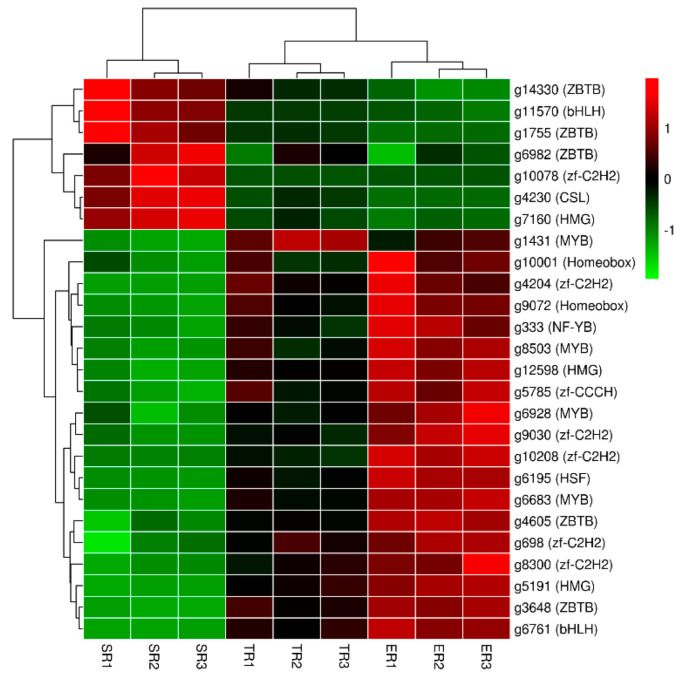
Heatmap of 26 Transcription factors in different stipe regions. Gene expression values (FPKM) were transformed to Z-score values.

As the master regulators control the expression of downstream genes, TFs have been demonstrated to have a critical role in mushroom fruiting body development [36]. To identify the transcription factors involved in stipe gradient elongation, about 26 TFs of 1409 DEGs were annotated, belonging to the bHLH, CSL, HMG, Homeobox, HSF, MYB, NF-YB, ZBTB, zf-C2H2, and zf-CCCH families (Figure S1). The heatmap in Figure 5 shows that all TFs were clustered into three main clades. In detail, about 18 TFs from 9 families had the highest expression in ER samples, followed by TR samples, and the lowest expression in SR samples. About 7 TFs of 3 ZBTB family genes, 1 bHLH family gene, 1 CSL family gene, 1 HMG family gene, and 1 zf-C2H2 family gene were upregulated in the stable region. Only 1 MYB family TF gene showed high expression in TR samples. These results suggest that a complicated gene network is needed to promote stipe elongation.

### 3.5. Gene Ontology (GO) and KEGG Pathway Enrichment Analysis of DEGs

**Figure 6:**
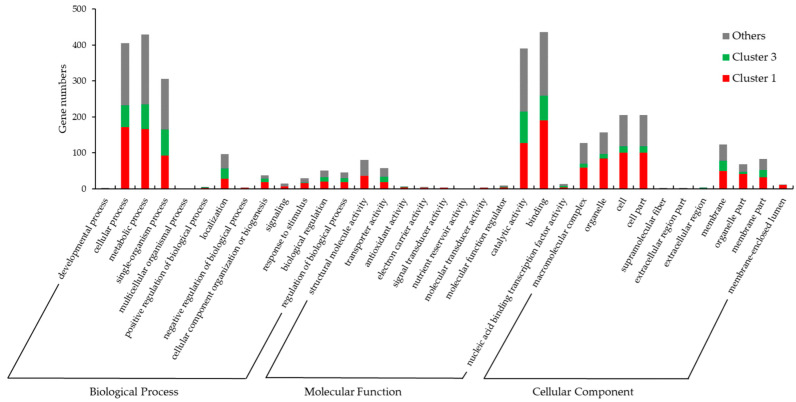
Gene Ontology (GO) classification of 1409 DEGs.

To define the functional annotation of DEGs, GO and KEGG pathway classification was carried out for the 1409 DEGs and the genes belonging to clusters 1 and 3. As shown in Figure 6 and Table S7, GO annotation was applied to 857 genes (about 60.8% of 1409 DEGs), which were assigned to 35 terms (level 2) in 3 categories. In detail, about 1429 items were enriched in the GO biological process category, mainly involved in metabolic process (430 GO items), cellular process (405 GO items), and single-organism process (306 GO items). About 1007 items were enriched in the GO molecular function category, mainly involved in binding (436 GO items), catalytic activity (391 GO items), structural molecule activity (81 GO items), and transporter activity (58 GO items). About 988 items were enriched in the GO cellular component category, mainly involved in cell (205 GO items), cell part (205 GO items), organelle (157 GO items), macromolecular complex (127 GO items), membrane (123 GO items), membrane part (83 GO items), and organelle part (68 GO items). The DEGs of cluster 1 (positively related to stipe elongation) and cluster 3 (negatively related to stipe elongation) showed different GO item enrichment. Compared with cluster 3, the genes in cluster 1 are mainly involved in macromolecular complex, organelle, cell, cell part, membrane, membrane part, organelle part, membrane-enclosed lumen, structural molecule activity and response to stimulus, cellular component organization or biogenesis terms.

**Figure 7:**
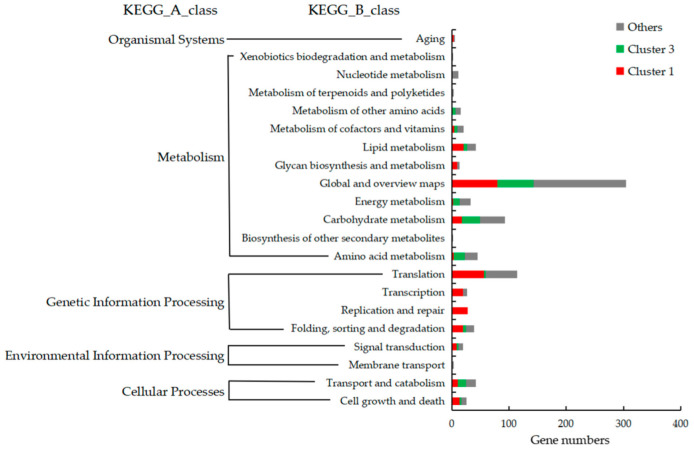
Kyoto Encyclopedia of Genes and Genomes (KEGG) classification of DEGs among different groups.

As shown in Figure 7 and Table S8, 684 genes (about 48.5% of the 1409 DEGs) mapped to 105 KEGG pathways, which can be classified into 5 groups and 21 subgroups, and are mainly enriched in the genetic information processing and metabolism classes. The genes involved in lipid metabolism, glycan biosynthesis and metabolism, translation, transcription, folding, sorting, and degradation pathways, and all genes involved in replication and repair pathway belong to cluster 1 DEGs. Cluster 3 DEGs are mainly involved in pathways of the metabolism class, including energy metabolism, carbohydrate metabolism, amino acid metabolism, and global and overview maps. The results indicate that cell replication and growth, which need DNA replication and cell component synthesis, may be the main factors promoting stipe elongation. The SR segment is the main region for nutrition and energy metabolism.

**Figure 8:**
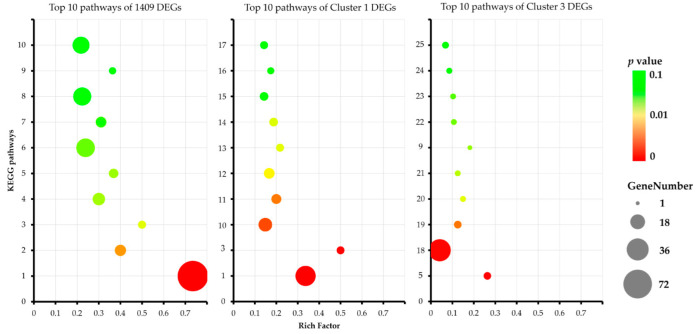
Bubble diagrams of top 10 enriched KEGG pathways of among different groups. Numbers on Y-axis represent KEGG pathways: 1. ribosome; 2. pentose phosphate pathway; 3. fatty acid elongation; 4. glycolysis/gluconeogenesis; 5. sulfur metabolism; 6. carbon metabolism; 7. citrate cycle (TCA cycle); 8. biosynthesis of amino acids; 9. ascorbate and aldarate metabolism; 10. spliceosome; 11. nucleotide excision repair; 12. mRNA surveillance pathway; 13. mismatch repair; 14. base excision repair; 15. DNA replication; 16. unsaturated fatty acid biosynthesis; 17. 2-oxocarboxylic acid metabolism; 18. metabolic pathways; 19. pyruvate metabolism; 20. galactose metabolism; 21. nicotinate and nicotinamide metabolism; 22. alanine, aspartate, and glutamate metabolism; 23. fructose and mannose metabolism; 24. valine, leucine, and isoleucine degradation; 25. ubiquitin-mediated proteolysis. Rich factor represents the ratio between DEGs and all annotated genes enriched in the pathway. Bubble scale represents the number of different genes, and intensity of bubble color represents adjusted *p*-value.

The top 10 enrichment pathways of clusters 1 and 3 and the 1409 DEGs are shown in Figure 8 and Table S8. The bubble diagrams suggest that the pathways of ribosome (*p*-value 3.15 × 10^−36^, rich factor 0.73), pentose phosphate pathway (*p*-value 5.98 × 10^−3^, rich factor 0.4), and fatty acid elongation (*p*-value 1.79 × 10^−2^, rich factor 0.5) are significantly enriched in the 1409 DEGs. In addition, ribosome (*p*-value 4.62 × 10^−16^, rich factor 0.34) and fatty acid elongation (*p*-value 2.69 × 10^−4^, rich factor 0.5) are also enriched in cluster 1 genes; sulfur metabolism (*p*-value 1.26 × 10^−4^, rich factor 0.26), metabolic pathways (*p*-value 4.99 × 10^−4^, rich factor 0.04), pyruvate metabolism (*p*-value 4.53 × 10^−3^, rich factor 0.13), galactose metabolism (*p*-value 1.70 × 10^−2^, rich factor 0.15), and some other metabolism pathways are enriched in cluster 3 genes. The results indicate that the ribosome and fatty acid elongation pathways may play an important positive role related to stipe elongation.

### 3.6. Ribosome Pathway Gene Expression Patterns Are Consistent with Stipe Elongation Rate

**Figure 9:**
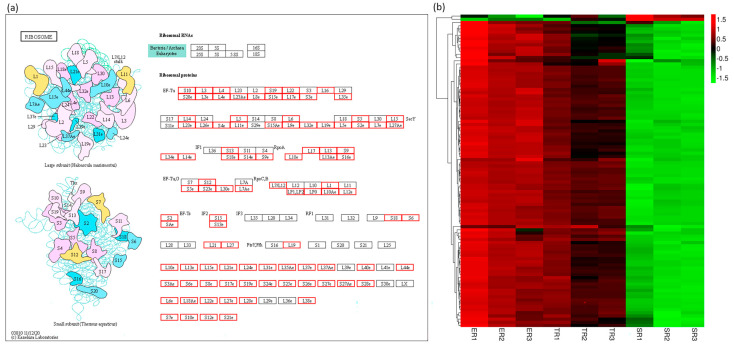
Differentially expressed genes of ribosome pathway. (**a**) Putative ribosome pathway in *F. filiformis* generated by KEGG analysis. Boxes with a black border mean no genes were annotated, boxes with a red border mean at least one gene was annotated. (**b**) Heat map showing expression profiles of ribosome genes. Gene expression values (FPKM) were transformed to Z-score values.

Ribosomes, as important ribonucleoprotein complexes, are responsible for translating mRNA into protein and essential for cell viability [37]. Variations in the level of ribosomes can affect cell fate and developmental transitions in tissues [38]. To further investigate the ribosome pathway gene expression patterns of different stipe regions, all 13,128 expressed genes were mapped onto the ribosome pathway. As shown in Figure 9a and Table S9, 98 genes were mapped to 97 proteins in the Ribosome pathway. The heat map of gene expression patterns shows that the genes were clustered into two main clades (Figure 9b). The bigger clade included 96 genes (33 genes belonging to cluster 1 DEGs), and the gene expression was highest in ER samples, followed by TR samples, and lowest in SR samples. Our results provide evidence that ribosome gene expression is consistent with the elongation rate of different stipe regions, which may be involved in stipe gradient elongation.

### 3.7. Long-Chain Fatty Acid Synthesis Pathway Involved in Stipe Gradient Elongation and Regulated by NADPH Oxidase-Derived ROS Signaling Molecules

**Figure 10:**
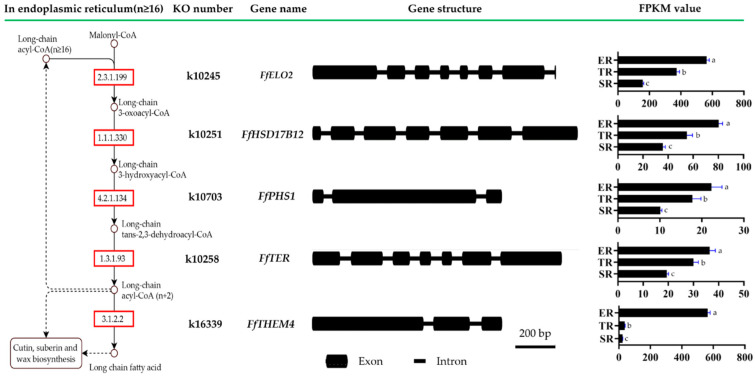
Long-chain fatty acid synthesis of fatty acid elongation pathway (PATH: map00062, Module: M00415) gene structure and expression pattern based on FPKM values of RNA-seq data. Letters over columns denote significant differences (*p* < 0.05, *n* = 3, paired *t*-test).

**Figure 11:**
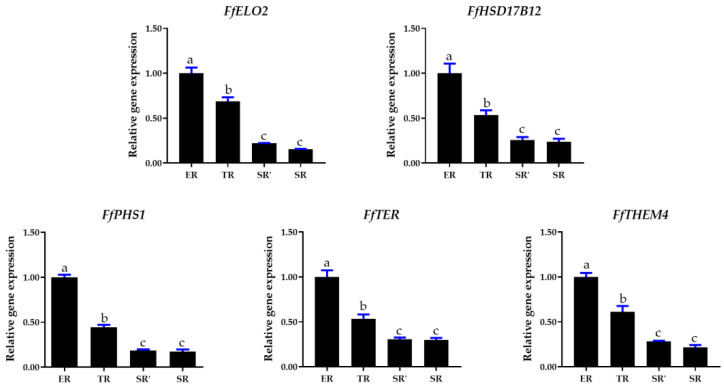
Expression of long-chain fatty acid synthesis of fatty acid elongation pathway (PATH: map00062, Module: M00415) genes in elongation region (ER), transition region (TR), and two stable regions (SR’ and SR). Letters over columns denote significant differences (*p* < 0.05, *n* = 4, paired *t* test).

Fatty acids are essential for membrane biosynthesis in all organisms. It was reported in plants that saturated very-long-chain fatty acids could promote cotton fiber and *Arabidopsis* cell elongation [39]. The KEGG pathway enrichment result showed that all genes mapped to long-chain fatty acid synthesis of the fatty acid elongation pathway (PATH: map00062, Module: M00415) belonged to cluster 1 DEGs (Table S8). For further investigation of long-chain fatty acid synthesis pathway gene expression patterns, five key enzymes encoding genes of *FfHSF17B12*, *FfPHS1*, *FfTER*, *FfTHEM4*, and *FfELO2* were annotated, and the sequences were submitted to NCBI after confirmation by Sanger sequencing (GenBank ID numbers OP822038-OP822042). The gene structures and FPKM values of ER, TR, and SR samples are shown in Figure 10. All five genes showed the same expression patterns, which was highest in the ER group and lowest in the SR group. To further confirm the relationship between the gene expression of the long-chain fatty acid synthesis pathway and the stipe elongation rate, the related expression of the five genes was detected by qRT-PCR using samples of elongation region (ER), transition region (TR), and two stable regions (SR’ and SR). As shown in Figure 11, *FfHSF17B12*, *FfPHS1*, *FfTER*, *FfTHEM4*, and *FfELO2* genes had the same expression pattern in four stipe regions; all genes showed the highest expression in ER samples, followed by TR samples, and the lowest in SR’ and SR samples. Pearson’s correlation coefficient analysis showed that the expression patterns of *FfHSF17B12*, *FfPHS1*, *FfTER*, *FfTHEM4*, and *FfELO2* genes were significantly positively correlated with stipe elongation rates (Table S9).

**Figure 12:**
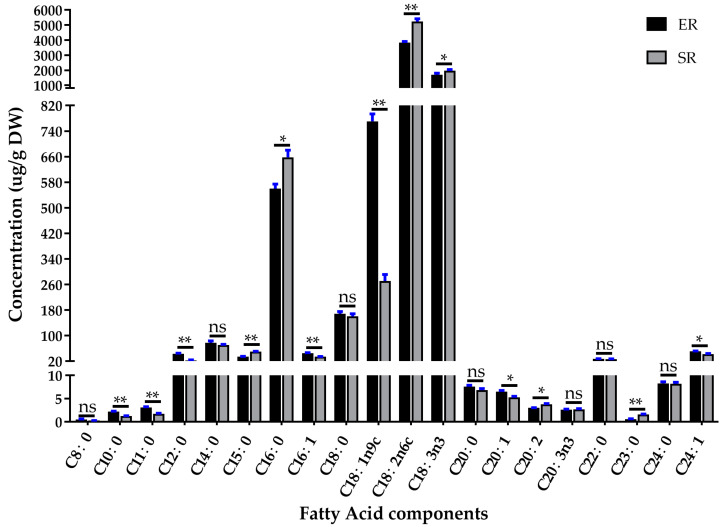
Fatty acid components of elongation region (ER) and stable region (SR). Significance levels were calculated by paired *t*-test (*n* = 4), * *p* < 0.05, ** *p* < 0.01, ns: no significance.

The fatty acid composition of elongation and stable regions is shown in Figure 12. Short-chain (C8 to C12), long-chain (C14 to C20), and very-long-chain (C20 to C24) fatty acid were distributed in both elongation and stable regions of the stipe. However, the concentrations were significantly different. Specifically, the saturated fatty acids C10:0, C11:0, and C12:0 and unsaturated fatty acids C16:1, C18:1n9c, C20:1, and C24:1 were significantly higher in ER than in SR; on the contrary, the saturated fatty acid C15:0, C16:0, C20:2, and C23:0 and unsaturated fatty acids C18:2n6c and C18:3n3 were significantly lower in ER than SR. Notably, the concentration of oleic acid (C18:1n9c) in ER was about 2.8 times that in SR. In *Caenorhabditis elegans*, it was demonstrated that the *ELO2* gene acts as a key enzyme during C18:1n9c synthesis from C16:0 [40]. Here, we found that the concentration of C16:0 was lower and C18:1n9c was higher in the elongation region, which was consistent with the expression pattern of the *FfELO2* gene. All of the above results suggest that the upregulation of long-chain fatty acid synthesis pathway genes, including *FfELO2* in the elongation region, may be involved in C18:1n9c synthesis and have a positive relationship with the stipe elongation rate.

**Figure 13:**
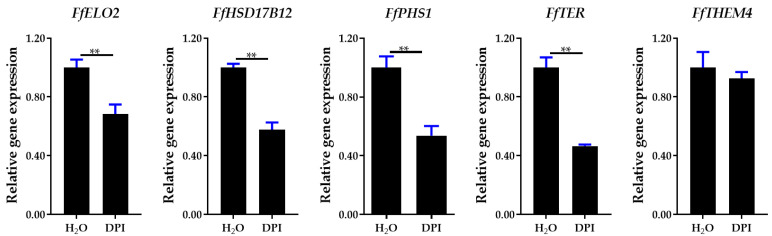
Expression of long-chain fatty acid synthesis of fatty acid elongation pathway (PATH: map00062, Module: M00415) genes in DPI and water treatment samples. Significance levels were calculated by *t*-test (*n* = 4), ** *p* < 0.01.

**Figure 14:**
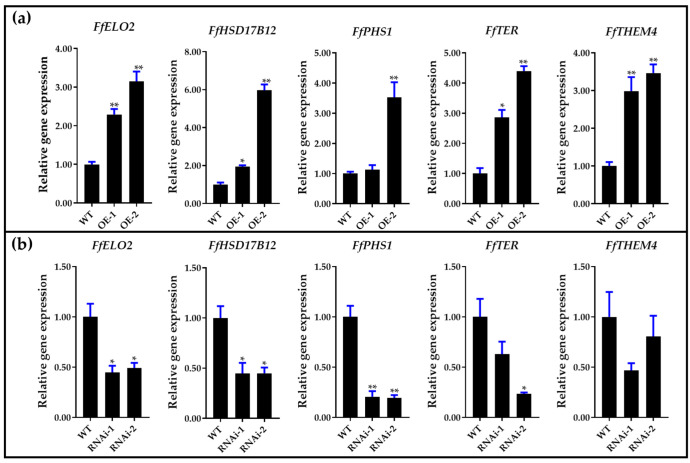
Expression of long-chain fatty acid synthesis of fatty acid elongation pathway (PATH: map00062, Module: M00415) genes in wild-type (WT) and *FfNoxA*: (**a**) overexpression lines and (**b**) RNAi lines. Significance levels were calculated by paired *t*-test compared with WT sample (*n* = 3), * *p* < 0.05, ** *p* < 0.01.

Our previous results demonstrated that ROS generated by NADPH oxidase act as key signaling molecules in regulating stipe gradient elongation [6]. To study the relationship between NADPH oxidase-derived ROS signaling and long-chain fatty acid synthesis, the gene expression patterns were detected in samples treated with diphenyleneiodonium chloride (DPI, an NADPH oxidase inhibitor) [41]. As shown in Figure 13, DPI treatment downregulated the gene expression of the long-chain fatty acid synthesis pathway, and the levels of the *FfHSF17B12*, *FfPHS1*, *FfTER*, and *FfELO2* genes were significantly inhibited. In addition, the overexpression and RNA interference mutants of *FfNoxA* (membrane-bound catalytic subunit of NADPH oxidase encoding gene) generated by our previous study were also used for detecting the expression of five genes in the long-chain fatty acid synthesis pathway [6]. The results in Figure 14 show that all five genes were upregulated in *FfNoxA* overexpressing strains, and four of them were significantly downregulated in *FfNoxA* RNAi strains (except for *FfTHEM4*). These results provide evidence that the long-chain fatty acid synthesis pathway may be regulated by NADPH oxidase-derived ROS signaling.

## 4. Discussion

Stipe gradient elongation is a common phenomenon in many basidiomycete fungi, such as *F. velutipes*, *C. cinerea*, and *A. bisporus* [3,4,5]. It is commonly accepted that stipe elongation growth is mainly attributed to manifold cell elongation rather than increased cell numbers [2]. Our study also shows that the stipe cell length significantly increased from the elongation region to the stable region, but was similar in two segments of the stable region (Figure 1c). Niu et al. suggested that the stipe cell wall architecture varies in elongation and non-elongation regions, and the thickness of stipe cell wall is inversely proportional to the cell elongation rate [8]. It was also demonstrated that several proteins and signaling pathways could mediate stipe cell extension and are involved in stipe elongation [5,6,10,13,14]. However, the stipe is usually treated as an entire organization in most transcriptome studies [42,43], and the transcriptional landscape of stipe gradient elongation remains to be clarified.

In this study, we provide a global view of stipe gradient elongation at the transcriptional level based on the RNA-seq method. We found that gene expression showed a high correlation between ER and TR samples (Figure 2), although the stipe elongation rate and cell length were significantly different (Figure 1b,c). The result suggests that the ER and TR may have a similar bio-process except for a different elongation ability. On the contrary, the gene expression in SR samples showed high variety in ER and TR samples (Figure 2). The STEM analysis of 1409 DEGs also showed that more than 1300 genes (including 857 downregulated and 553 upregulated genes) in clusters 1 to 4 had significantly different expression in SR samples compared with ER and TR samples (Figure 4a). These results indicate that bio-processes occurring in the stable region are different from those in ER and TR segments.

Some research has suggested that genes related to the cell wall synthesis are very important for the cell growth and thus cell elongation [9,10,11,12]. According to our transcriptome data, 7 glucanase genes, 14 chitinase genes, 2 glucan synthase genes, and 11 chitin synthase genes were annotated, and the FPKM value of these genes were shown in Table S10. For the most part, genes of glucanase, glucan synthase and chitin synthase showed the lowest expressed in SR segment, but the difference expression patterns were found in different in chitinase genes. These results suggested that the genes of cell wall synthesis, especially glucanase, glucan synthase, and chitin synthase genes, are involved in stipe elongated regulation.

Kamada summarized the early studies and suggested that stipe elongation is not a simple expansion of water intake, but a process involving the transmission of nutrients and accumulation of dry matter [44]. Here, we found that about 50% of GO items in the cellular component category (Figure 6), about 60% of KEGG items in the genetic information processing class (Figure 7), and about 98% of ribosome pathway mapped genes (Figure 9) were expressed consistent with the elongation rate of different regions, which provides evidence that the stipe elongation process is not only a synthesis of cellular components, but also the transmission and replication of genetic information.

**Figure 15:**
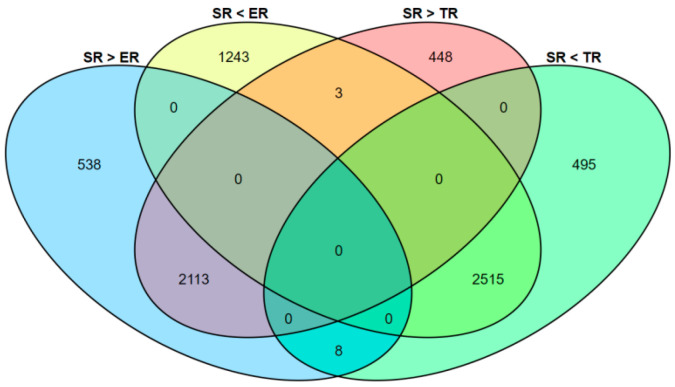
Venn diagram showing DEGs of SR samples compared with ER and TR groups. ER, elongation region; TR, transition region; SR, stable region.

Few studies have focused on the stable (non-elongation) region of stipe, since the stable region does not elongate. According to our RNA-seq data, about 2515 DEGs had the lowest expression in SR samples but 2113 DEGs had the highest expression (Figure 15). Further analysis of the 2113 upregulated DEGs in SR showed about 2070 GO items enriched in the biological process category, 1385 GO items enriched in the molecular function category, and 736 GO items enriched in the cellular component category. About 2826 GO items were enriched in the top five level 2 GO terms of catalytic activity, binding, metabolic process, cellular process, and single-organism process (Figure S2, Table S7). The KEGG pathway annotation of the 2113 DEGs also showed about 1141 DEGs enriched in 82 pathways of the KEGG A metabolism class. About 654 genes were enriched in the top five KEGG B classes: global and overview maps (310 genes), carbohydrate metabolism (114 genes), amino acid metabolism (101 genes), transport and catabolism (76 genes), and translation (53 genes) (Figure S3, Table S8). These results suggest that the complex bio-processes of nutrient metabolism, energy metabolism, substance transportation, and amino acid biosynthesis happen in the stable region.

Qin et al. (2007) demonstrated that saturated very-long-chain fatty acids (C24:0) could activate ethylene biosynthesis, promoting cotton fiber and *Arabidopsis* cell elongation [40]. Several studies also found that 10-oxo-trans-8-decenoic acid (ODA) synthesis from linoleic acid (C18:2n6c) could stimulate stipe elongation in *Agaricus bisporus* [45,46]. ODA was also demonstrated to stimulate the mycelial growth of Yun Chih (*Trametes versicolor*) and winter mushroom (*Flammulina filiformis*) on agar plates [47]. Our result shows that a fatty acid elongation pathway (PATH: map00062, Module: M00415) related to oleic acid (C18:1n9c) synthesis may be involved in stipe elongation and may be regulated by NADPH oxidase-derived ROS signaling molecules. Based on the similar chemical structure of oleic acid and linoleic acid, we speculate that a high concentration of oleic acid may be a precursor for some active compounds and stimulate stipe elongation. However, more studies should be carried out to determine the relationship between oleic acid and stipe elongation.

## Data Availability

All RNA-seq data in this study are available in the National Center for Biotechnology Information Sequence Read Archive, under accession number PRJNA901539 (https://www.ncbi.nlm.nih.gov/sra/PRJNA901539, accessed on 15 November 2022). The other data are contained within the article or Supplementary Materials.

## Supplementary Materials

The following supporting information can be downloaded at https://www.mdpi.com/article/10.3390/jof9010064/s1: Figure S1. Family classification of differently expressed Transcription factors. Figure S2. Gene ontology (GO) classification of upregulated DEGs in SR. Figure S3. Kyoto Encyclopedia of Genes and Genomes (KEGG) classification of upregulated DEGs in SR. Table S1. Primers used in PCR. Table S2. Primers used in qRT-PCR. Table S3. Quality statistics of clean sequencing data. Table S4. FPKM values of 13,128 genes. Table S5. Total of 1409 significantly different DEGs among ER, TR, and SR groups. Table S6. Mean FPKM values of clusters 1 and 3 DEGs. Table S7. GO functional classification of DEGs. Table S8. KEGG functional classification of DEGs. Table S9. Pearson’s correlation coefficient analysis of expression patterns and stipe elongation rates of five genes. Table S10. FPKM values of genes related to the cell wall synthesis.
