# Supplementary material for: Transcriptome Profiling Reveals Candidate Genes Related to Stipe Gradient Elongation of Flammulina filiformis
Source: J Fungi (Basel). 2022 Dec 31;9(1):64. doi: 10.3390/jof9010064 (PMC9862757; doi:10.3390/jof9010064)
Supplement: Supplementary file 1 [file jof-09-00064-s001.zip › Table S1. Primers used in PCR.pdf]

Table S1 Primers used in PCR

Gene	Forward sequence（5'-3'）	Reverse sequence（5'-3'）
FfELO2	TGTCAAGCGAAAGAAGATCA	CCCACTATGTGTCCAGAAAA
FfHSD17B12	ACAAGTATCTCTGATTCCGC	GTACTTCTATGACGTGGACC
FfPHS1	TTTAAGCACCGTCGAAGTAA	CGAAGTACATCAGTGGTCAT
FfTER	AGAAAGGACCAAGGAATGAC	GTACTATGGTACGCTCGTTT
FfTHEM4	ACGCCTACCGATACATAAAC	TGGAAACGGTCTTGAGTAAG
