# Supplementary material for: Transcriptome Profiling Reveals Candidate Genes Related to Stipe Gradient Elongation of Flammulina filiformis
Source: J Fungi (Basel). 2022 Dec 31;9(1):64. doi: 10.3390/jof9010064 (PMC9862757; doi:10.3390/jof9010064)
Supplement: Supplementary file 1 [file jof-09-00064-s001.zip › Table S2. Primers used in qRT-PCR.pdf]

Table S2 Primers used in qRT-PCR

Gene	Forward sequence（5'-3'）	Reverse sequence（5'-3'）
FfELO2	CTTGCTGCTGCTCGTGCTCAT	AATGCGGTGGCGGAATGATGG
FfHSD17B12	CCGAGGAAGTCAAGCAGCACAA	CAGGAAGTGGAACTAGCAGGTTAGG
FfPHS1	GCCTTCCTCAACTACGCTACTCTC	TTTGCCTTGGAACCCTTGGATGG
FfTER	CCCGTTTCCCACCTAAAGTCAAAGA	GTCTATCTCTCCGCCAGCACCTAA
FfTHEM4	GGTCACTGCGACGCTGAACTTG	CCGCCTCCGCTATAGTCTTCTTGT
FfGPD	CCTCTGCTCACTTGAAGGGT	GCGTTGGAGATGACTTTGAA
FfRAS	TCAATGCGACGAGTAAAGAGAGG	CATAGGTCCCACATCTACATTTCG
