# Supplementary figures and images for: Transcriptome Profiling Reveals Candidate Genes Related to Stipe Gradient Elongation of Flammulina filiformis
Source: J Fungi (Basel). 2022 Dec 31;9(1):64. doi: 10.3390/jof9010064 (PMC9862757; doi:10.3390/jof9010064)

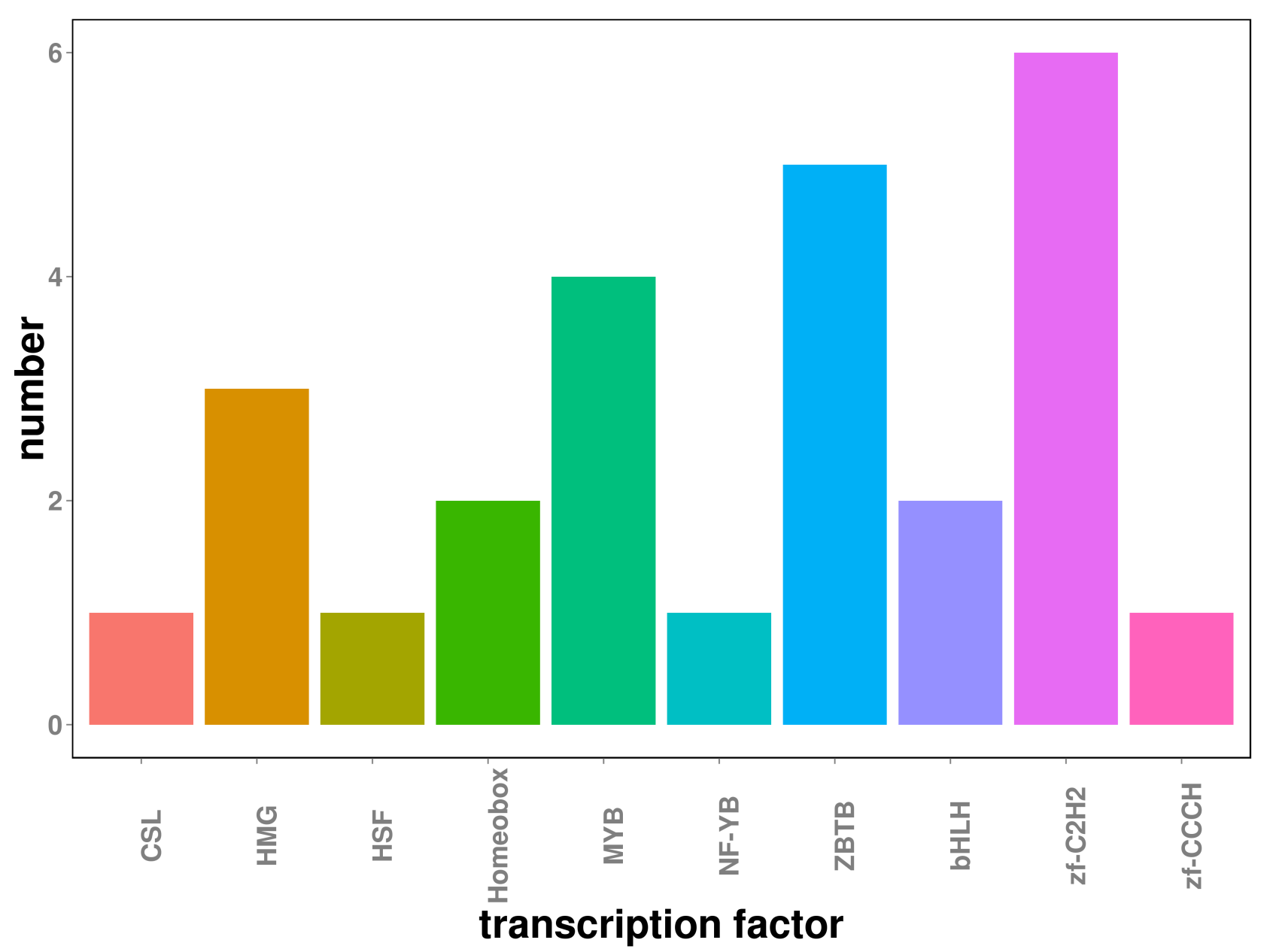

Supplement: Supplementary file 1 [file jof-09-00064-s001.zip › Figure S1. Family classification of differently expressed Transcription factors.png]

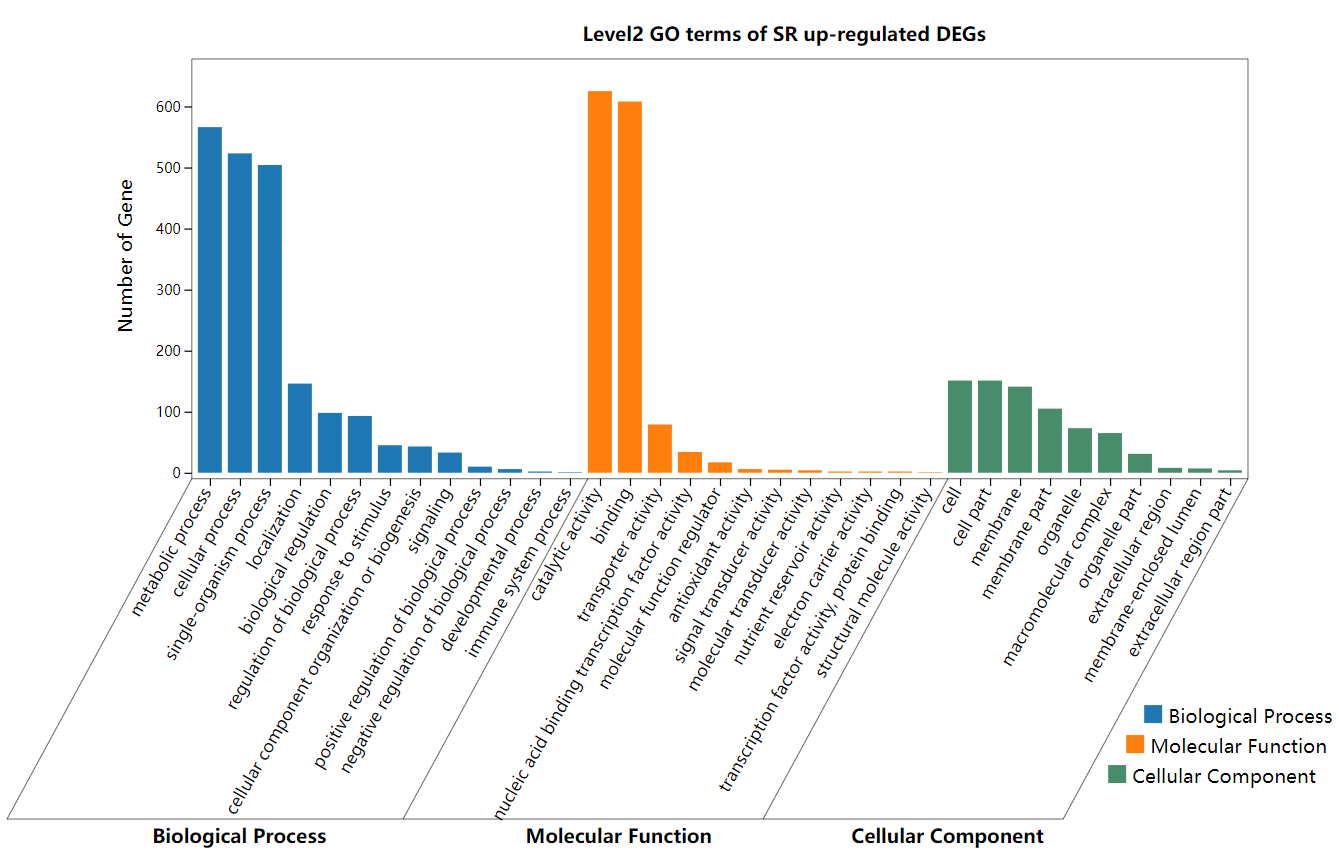

Supplement: Supplementary file 1 [file jof-09-00064-s001.zip › Figure S2. Gene ontology (GO) classification of upregulated DEGs in SR.png]

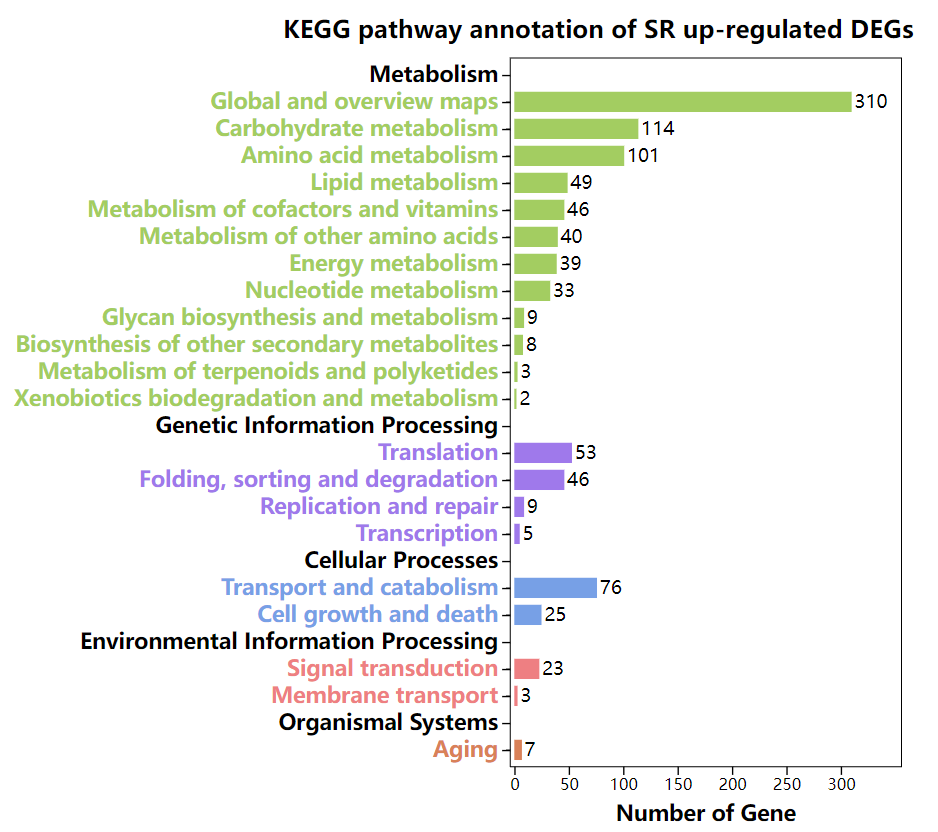

Supplement: Supplementary file 1 [file jof-09-00064-s001.zip › Figure S3. Kyoto Encyclopedia of Genes and Genomes (KEGG) classification of upregulated DEGs in SR.png]
